# Optimization of ultrasonic-assisted extraction of total flavonoids from *Zanthoxylum bungeanum* residue by response surface methodology and evaluation of its algicidal properties

**DOI:** 10.3389/fmicb.2025.1540631

**Published:** 2025-03-13

**Authors:** Jie Cheng, Long Tan, Yucai Wang, Mengwei Gao, Feifei Liu, Qi Wang, Chengshuai Xu, Chaobo Zhang, Wei Xu, Yuyong Hou, Tong Jiang, Lei Zhao

**Affiliations:** ^1^State Key Laboratory of Macromolecular Drugs and Large-scale Preparation, School of Pharmaceutical Sciences and Food Engineering, Liaocheng University, Liaocheng, China; ^2^State Key Laboratory of Engineering Biology for Low-carbon Manufacturing, Tianjin Institute of Industrial Biotechnology, Chinese Academy of Sciences, Tianjin, China; ^3^College of Agriculture and Biology, Liaocheng University, Liaocheng, China

**Keywords:** *Zanthoxylum bungeanum* residue, flavonoids, photosynthetic activity, allelopathic mechanism, *Tetrodesmus obliquus*

## Abstract

*Zanthoxylum bungeanum* residue has attracted increasing attention owing to its antibacterial effect, which presents potential as novel antimicrobial agents for the management of algal blooms. In this study, the ability of *Z. bungeanum* residue extract to control algal blooms has been firstly verified. Then, the response surface methodology was employed to optimize flavonoids yield, the primary antimicrobial component in extract, and the underlying photosynthetic inhibition mechanisms of extract on *Tetrodesmus obliquus* was investigated. Results showed that the highest yield of total flavonoids was increased to 4.08% when the extraction conditions were a liquid-to-solid ratio of 10:1, ethanol concentration of 60%, extraction temperature of 80°C, and extraction time of 30 min. Meanwhile, treatment with *Z. bungeanum* residue extract at doses of 40.0 mg/L significantly decreased the Fv/Fm and PIabs values of *T. obliquus* by 24.36 and 88.87% at 50 h, respectively. The added extract induced damage at multiple levels of physiological and biochemical processes in algal cells, including reduced electron transport capability, disrupted energy transfer in photosystem II, disruption of OEC structure, and altered energy distribution in PSII reaction center. To our knowledge, this study was the first verification of *Z. bungeanum* residue’s algicidal potential, and these findings in our study contribute to a deeper understanding of the allelopathic mechanisms of *Z. bungeanum* residue extract and offer valuable insights for the management of algal blooms.

## Graphical abstract

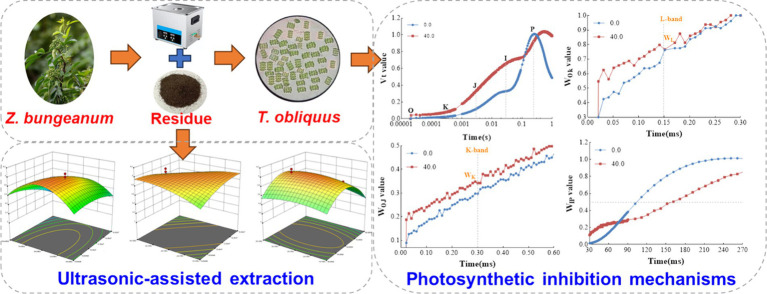

*Zanthoxylum bungeanum* residue extract induced damage at multiple levels of physiological and biochemical processes in algal cells, including reduced electron transport capability, impaired energetic connectivity between antenna and PSII reaction centers, disruption of OEC structure, and altered energy distribution in PSII reaction center.

## Highlights

- *Zanthoxylum bungeanum* residue extract was chosen for inhibitory experiment on *T. obliquus*.
- The inhibitory mechanism was explored for the possibility to practical application.
- 40 mg/L of extract significant decreased in maximum quantum yield of PSII.
- The electron transport capability was suppressed in treated cells.
- Extract inhibited the energetic connectivity between antenna and PSII reaction centers.

## 1 Introduction

Rising environmental awareness has amplified worries about eutrophication’s negative impact on quality of life, leading to greater focus on water purification. Eutrophication-induced algal blooms present substantial risks to aquatic ecosystems, resulting in a series of challenges related to the economy, food safety, and public health (Wang F. et al., 2022; Zhu et al., 2014). These blooms, frequently driven by nutrient runoff from agricultural and urban sources, can lead to oxygen depletion in aquatic environments, creating hypoxic conditions that adversely affect fish and other aquatic organisms. Concurrently, the economic repercussions are significant, as both the fisheries and tourism sectors experience negative impacts due to the deterioration of water quality and the consequent esthetic decline of natural landscapes (Wang L. et al., 2024).

To address these challenges, implementing effective management strategies is crucial. Researchers employ physical, chemical, and biological methods to manage water blooms. Physical methods, such as UV treatments and photocatalytic reactions, have demonstrated high efficacy in degrading harmful compounds associated with these blooms (Wang L. et al., 2024). However, these methods are often time-consuming and labor-intensive (Zhang et al., 2013). Although chemical treatments can inhibit algal growth, they pose significant ecological safety risks and are prone to causing secondary pollution (Zhang et al., 2013). Consequently, tackling the challenges posed by algal blooms requires a multifaceted approach that considers ecological, economic, and health perspectives. This approach ensures the sustainability of water resources for future generations (Chen et al., 2023).

It is anticipated that the biological approach will evolve into a highly valuable algicidal technology. Studies have indicated that the application of allelochemicals sourced from macrophytes represents a promising strategy for managing algal proliferation. Several systematic reviews have highlighted that numerous macrophyte species exhibit a spectrum of effects on bloom-forming algae and cyanobacteria, with some demonstrating inhibitory properties that could be leveraged for ecological restoration efforts (Cheng K. et al., 2024; Wang and Liu, 2023). These compounds are not only biodegradable and environmentally benign but also demonstrate considerable potential in alleviating the detrimental impacts of algal blooms (Hilt and Gross, 2008). The mechanism by which these allelochemicals act against algae is primarily associated with the disruption of cellular structures and alterations in physiological and biochemical properties, including oxidative stress, programmed cell death, photosynthetic processes, and protein synthesis (Sun et al., 2021; Wang and Liu, 2023; Xu et al., 2022). Altogether, the allelopathic properties of aquatic macrophytes offer a promising strategy for the management of algal populations and the restoration of ecological equilibrium in freshwater ecosystems. Continued research into the specific allelochemicals involved and their mechanisms of action will be crucial for developing effective management strategies against harmful algal blooms.

*Zanthoxylum bungeanum* is an economic crop that serves as a source of oil, spice, and medicinal components, and is esteemed not only for its culinary applications but also for its notable medicinal properties (Zheng et al., 2022). The cultivation of *Z. bungeanum* is vital to agriculture in regions like Sichuan, significantly contributing to local cuisine, especially in dishes known for their unique numbing flavor (Bao et al., 2023). In China, annual output of *Z. bungeanum* exceeds 500,000 tons, and substantial quantities of waste are generated during the planting, harvesting, and processing of *Z. bungeanum* (Liang et al., 2024). These by-products, specifically *Z. bungeanum* residue and leaves, are generally discarded. In response to this issue, researchers have initiated investigations into converting this agricultural waste into valuable resources. Recent studies have underscored the importance of various bioactive compounds found in *Z. bungeanum*, with a focus on their pharmacological and toxicological properties (Zhang et al., 2017). Notably, *Z. bungeanum* residue exhibits functional components akin to those of *Z. bungeanum*. However, there has been limited research on the algal inhibition properties of these functional components from *Z. bungeanum* residue.

*Tetrodesmus obliquus*, a prevalent green alga, frequently coexists with cyanobacteria, contributing to mixed algal bloom formation (Amorim and do Nascimento Moura, 2021; Qian et al., 2019). This study verified the ability of *Z. bungeanum* residue extract to control algal blooms. Then, the ultrasonic-assisted extraction process of total flavonoids from *Z. bungeanum* residue was examined using response surface methodology. Finally, the research elucidated the allelopathic inhibitory effects of the improved ultrasonic-assisted total flavonoid extract from *Z. bungeanum* residue on the photosynthetic activity of *T. obliquus*. These findings enhance the understanding of the antimicrobial mechanisms of *Z. bungeanum* residue extract and provide valuable insights for managing algal blooms.

## 2 Materials and methods

### 2.1 Materials and reagents

*Tetrodesmus obliquus* (FACHB-417) was obtained from the Freshwater Algae Culture Collection at the Institute of Hydrobiology of the Chinese Academy of Sciences. The algae were initially cultured in sterilized BG-11 medium, prepared by dissolving 1.7 grams of BG-11 powder in 1000 mL of purified water within a 1 L conical flask. The algal cultures were kept in a photo-shaking incubator under controlled conditions: a temperature of 25 ± 1°C, a light intensity of 6,000 lux, a rotation speed of 130 rpm, and a photoperiod of 14 h light and 10 h dark.

*Zanthoxylum bungeanum* residue was obtained from Chongqing Fuliang Grain and Oil Co., Ltd., China, and subsequently pulverized using a high-speed pulverizer before being dried in an oven at 60°C until a constant weight was achieved. The *Z. bungeanum* residue was subjected to sieving using screens of varying mesh sizes: 40 mesh (aperture size 0.406 mm), 60 mesh (aperture size 0.251 mm), and 140 mesh (aperture size 0.106 mm), resulting in the production of *Z. bungeanum* powders designated as Sample 1, Sample 2, and Sample 3, respectively. Subsequently, the sieved *Z. bungeanum* powders undergo a defatting process by immersion in petroleum ether for 24 h, utilizing a solvent-to-sample ratio of 7.5 mL of petroleum ether per 1.0 g of sample. Following defatting, vacuum filtration was employed to separate the *Z. bungeanum* powders from the organic solvent. The defatted powders are then dried in an oven at 60°C until a constant weight is achieved, after which they are sealed and stored for subsequent use.

### 2.2 Determination of total flavonoid yield

The total flavonoid content was expressed in terms of rutin equivalents, providing a standardized measure for comparison across different samples. Briefly, the process begins with the preparation of a series of rutin standard solutions at known concentrations. Subsequently, the resulting solutions were analyzed using the aluminum nitrate colorimetric technique, which involves the formation of a complex between flavonoids and aluminum ions (Cheng J. et al., 2024). These solutions are then subjected to spectrophotometric analysis, with absorbance measured at a specific wavelength of 510 nm. A standard curve was generated by plotting absorbance values against concentrations, and the total flavonoids concentration in *Z. bungeanum* residue was determined using the rutin standard curve. The total flavonoids yield was calculated as Y = (C × V)/W × 100%, where C shows the extract concentration (mg/mL), V denotes the extract volume (mL), and W represents the specimen mass (g).

### 2.3 Optimization of ultrasonic-assisted extraction of total flavonoids from *Zanthoxylum bungeanum* residue

The ultrasonic-assisted extraction process for enhancing total flavonoids yield from *Z. bungeanum* residue was optimized using single-factor optimization to preliminarily establish the extraction parameter range. This study examined the effects of four variables on the yield of total flavonoids from *Z. bungeanum* residue: liquid-to-solid ratio (5, 10, 15, 20, 25 mL/g), ethanol concentration (20, 40, 60, 80, 100%), extraction duration (25, 35, 45, 55, 65 min), and extraction temperature (40°C, 50°C, 60°C, 70°C, 80°C). The experimental approach employed a single-factor variable method combined with analysis of variance, systematically varying one factor while maintaining the others constant, and sequentially conducting experiments for each of the four variables. Single-factor experiments identified the liquid-to-solid ratio (X1, mL/g), ethanol concentration (X2, %), and extraction time (X3, min) as the primary factors influencing the yield of total flavonoids (Y, %) in *Z. bungeanum* residue. A Box–Behnken design was utilized to optimize the extraction of total flavonoids from *Z. bungeanum* residue, with the yield serving as the evaluation metric. Each variable was assigned three levels, coded as −1, 0, and + 1, to indicate low, medium, and high values, respectively (Supplementary Table S1).

### 2.4 Allelopathic inhibitory effect of *Zanthoxylum bungeanum* residue extracts

*Tetrodesmus obliquus* was transferred to 50 mL of sterilized BG11 medium in 250 mL conical flasks during the logarithmic growth phase, with an initial optical density at 680 nm (OD_680_) of 0.2. The experimental conditions aligned with those outlined in Section 2.1. Various volumes of *Z. bungeanum* residue extract solutions were introduced to these cultures, resulting in the concentrations of 0.0 and 40.0 mg/L. Each concentration was tested in triplicate. Fluorescence parameters are frequently indicative of the physiological status of a sample, including its growth, exposure to stress, and overall health or normal growth (Gajdosik et al., 2022). The allelopathic inhibitory effect of the extracts on algal blooms was evaluated by measuring chlorophyll a fluorescence transients with a chlorophyll fluorometer (AquaPen-C AP110-C, Photon Systems Instruments, The Czech Republic), and quantifying the JIP transients with the same cell density after exposure to *Z. bungeanum* residue extracts. Following a 20-min dark pre-conditioning, fluorescence parameters of the test samples were measured at designated time intervals (20, 30, and 50 h). To compare the changes in fluorescence parameters among the different treatments, we standardized the fluorescence values and analyzed them with the JIP test.

### 2.5 Data processing

The data were analyzed using univariate analysis of variance (ANOVA), and Duncan’s multiple comparison test. Statistical significance was established at a 5% level (*p* < 0.05). SPSS 19.0 statistical software (SPSS Inc., Chicago, USA) was utilized for data analysis, and diagrams were plotted with GraphPad Prism 9.0 (San Diego, CA, USA).

## 3 Results and discussion

### 3.1 Optimized ultrasonic-assisted extract process enhance total flavonoids yield

#### 3.1.1 Influence of liquid-to-solid ratio on total flavonoids yield in *Zanthoxylum bungeanum* residue

**Figure 1:**
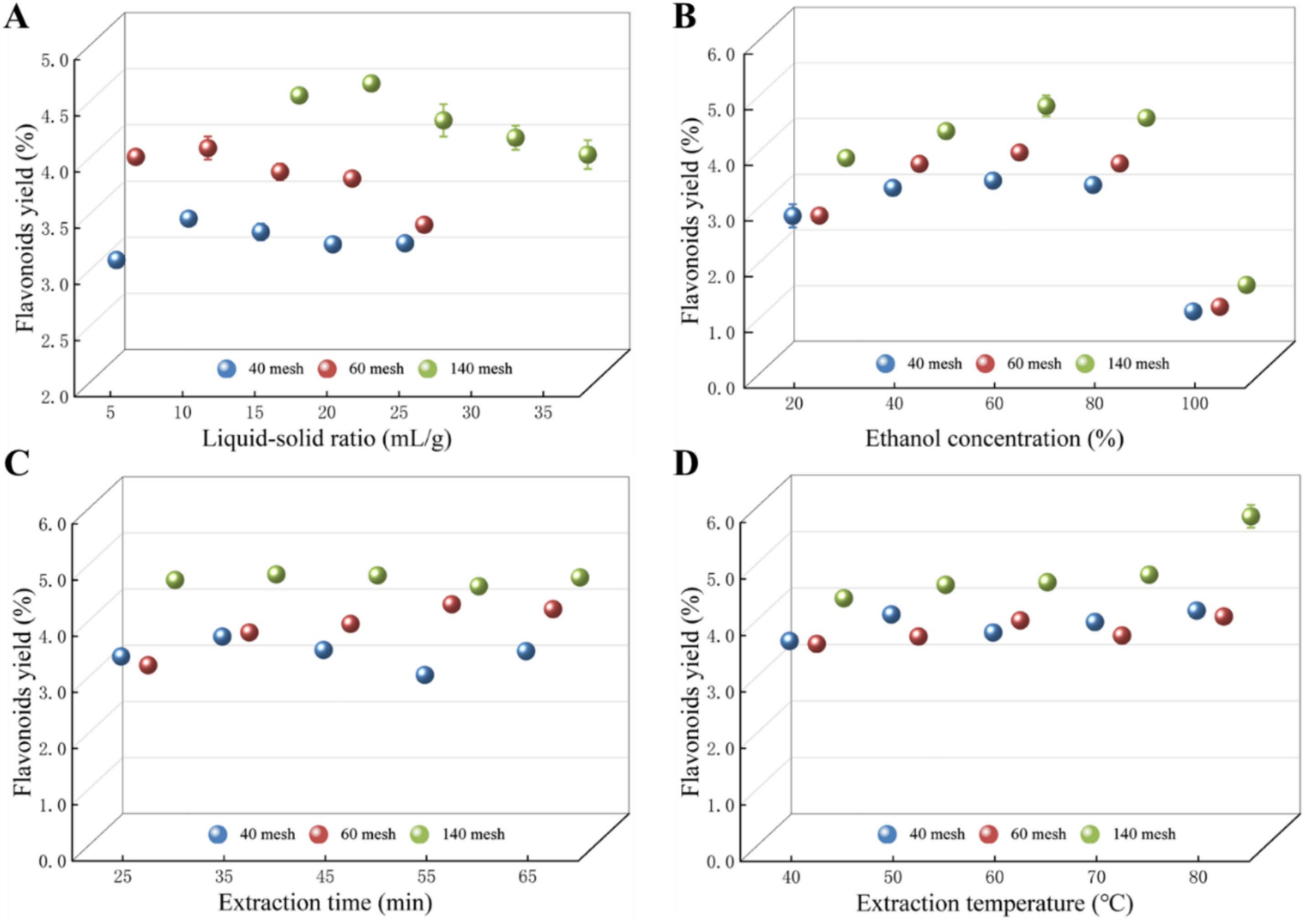
Effects of **(A)** liquid–solid ratio, **(B)** ethanol concentration, **(C)** extraction time, and **(D)** extraction temperature on the yield of total flavonoids in *Z. bungeanum* residue. Values represent the mean of three independent measurements and bars indicate SD. All processes were biologically repeated in three independent and parallel experiments.

A single-factor experiment examined the impact of four variables on total flavonoid yield, including liquid-to-solid ratio, ethanol concentration, extraction time, and extraction temperature (Figure 1). The liquid–solid ratio is a vital factor that can be fine-tuned to enhance the extraction process across different plant materials (Luo et al., 2018; Wu et al., 2022), and the effect of the liquid-to-solid ratio on the yield of total flavonoids from *Z. bungeanum* residue was investigated with a ratio ranging from 5:1 to 35:1 mL/g. As shown in Figure 1A, the total flavonoids yield in Sample 1 and Sample 2 significantly increased before the liquid–solid ratio reached 10:1 mL/g. After that, the total flavonoids yield changed slightly, and the total flavonoids yield started to decrease after the liquid–solid ratio exceeded 10:1 mL/g. And the maximum total flavonoids yield for Sample 3 appeared at a ratio of 20:1 mL/g. The above results indicated that adjusting the liquid–solid ratio can significantly affect the yield of flavonoids, and the similar tendency was also observed for the yield of total flavonoids from *Chionanthus retusa* leaves (Wang Z. et al., 2022).

#### 3.1.2 Effect of ethanol concentration on the yield of total flavonoids in *Zanthoxylum bungeanum* residue

Ethanol is an effective solvent for extracting bioactive compounds like flavonoids, as it can dissolve both polar and non-polar substances. The optimization of solvent mixtures for flavonoid extraction from *Phalaenopsis* leaves highlighted the importance of ethanol in the extraction process (Sanjaya et al., 2024). These findings emphasize that ethanol concentrations have proven to be a critical factor in the extraction of total flavonoids. Further research into optimizing ethanol concentrations for specific plant materials could enhance the extraction processes and improve the efficacy of the resulting bioactive compounds. In this study, the effects of ethanol concentrations (20, 40, 60, 80, 100%) on the extraction efficiency of total flavonoids were investigated (Figure 1B). Results indicated that the total flavonoids yield from various samples was sharply promoted when the ethanol concentrations ranged from 20 to 60%. And the yield of total flavonoids showed a downward trend after the ethanol concentrations exceeded 60%. The similar tendency was also observed in previous study, in which a 50% ethanol concentration has been reported to yield the highest total polyphenol and flavonoid content compared to 80 and 100% ethanol concentrations (Jung et al., 2023). Therefore, 60% ethanol concentration was chosen as the optimum extraction solvent of total flavonoids and used in subsequent experiments.

#### 3.1.3 Effect of extraction time on the yield of total flavonoids in *Zanthoxylum bungeanum* residue

The extraction time is also a pivotal factor that must be carefully optimized to maximize the yield of total flavonoids from plant materials, and numerous studies consistently indicate that shorter or longer extraction times can significantly affect the efficiency of flavonoid extraction, underscoring the importance of methodical optimization in extraction protocols (Li et al., 2024; Liao et al., 2021). In this study, the effect of extraction times (25, 35, 45, 55, 65 min) on the yield of total flavonoids was tested (Figure 1C). Within a certain time range, the yield of total flavonoids in *Z. bungeanum* residue showed an overall trend of increasing and then decreasing with the increase of extraction time. The maximum yields of total flavonoids in Sample 1, Sample 2, and Sample 3 were reached at extraction time of 35, 55, and 35 min, respectively. When the duration of ultrasonic treatment is short, the cell walls of the *Z. bungeanum* residue remain largely intact, inhibiting the complete release of flavonoid compounds and consequently diminishing the yield. Conversely, as the ultrasonic treatment is prolonged, the cellular structure undergoes more extensive disruption, facilitating an increased dissolution of total flavonoids from the residue and thereby enhancing the yield. However, if the ultrasonic treatment exceeds an optimal duration, the intense vibrations associated with the ultrasonic waves can inflict damage on the molecular structure of the flavonoids, ultimately leading to a reduction in yield. Our research is consistent with previous findings, in which the optimal extraction time of enzyme-assisted ultrasonic extraction of total flavonoids from *Abelmoschus manihot* residue was found to be 40 min, resulting in a yield of 3.46 ± 0.012% (w/w) total flavonoids when specific enzyme concentrations and conditions were added (Chu et al., 2024).

#### 3.1.4 Effect of extraction temperature on yield of total flavonoids in *Zanthoxylum bungeanum* residue

The extraction temperature plays a crucial role in determining the yield of total flavonoids from various plant materials. Several studies have reported the positive correlation between elevated extraction temperature and extraction efficiency of flavonoids (Li et al., 2024; Liao et al., 2021; Mai et al., 2020; Vieira et al., 2019), and the evidence suggests that careful control of extraction temperature is fundamental for maximizing the yield of total flavonoids across various extraction methods and plant sources (Chu et al., 2024; Yang et al., 2017). In this study, the effect of extraction temperature (40, 50, 60, 70, and 80°C) on the yield of total flavonoids was investigated, and there was a significant improvement in total flavonoids yield as the extraction temperature increased from 40°C to 80°C (Figure 1D). The cavitation phenomenon induced by ultrasonic waves enhances the interaction among internal compound molecules, thereby facilitating the rapid dissolution of flavonoid active components within the solvent. At lower ultrasonic temperatures, the thermal energy is inadequate to effectively promote the release and dissolution of total flavonoids, leading to a reduced yield. Conversely, as the ultrasonic temperature rises, the thermal motion energy of the compound molecules in the extraction medium increases, resulting in an accelerated diffusion rate. This, in turn, enhances the solubility of flavonoid compounds in the solvent, thereby improving the yield of total flavonoids (Chen et al., 2018).

### 3.2 Model fitting and response surface analysis

**Figure 2:**
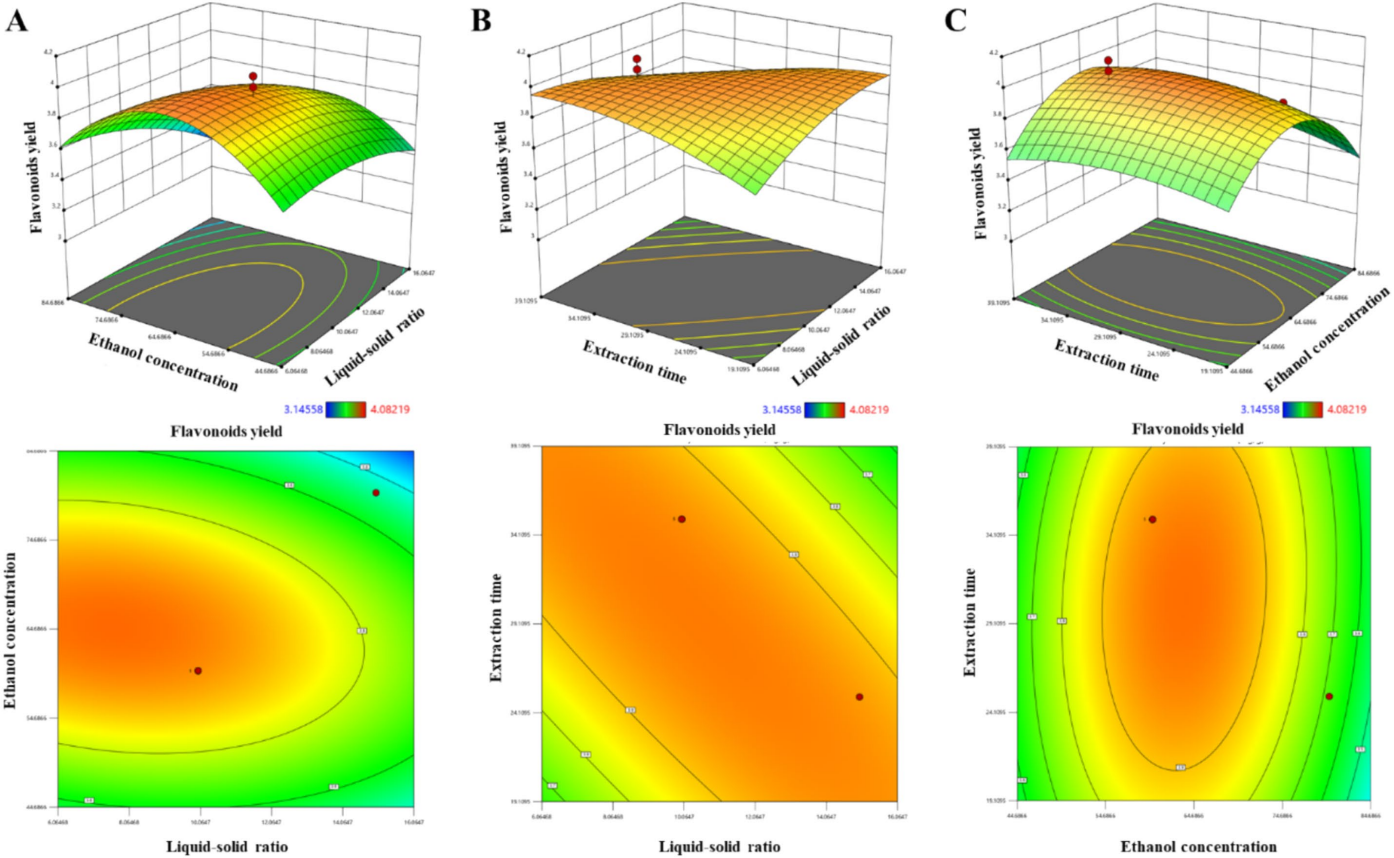
The three-dimensional response surfaces and two-dimensional contour plots showing the effects of liquid–solid ratio, ethanol concentration, and extraction time on the extraction efficiency of total flavonoids in *Z. bungeanum* residue. **(A)** The impact of the interactive effects between liquid–solid ratio and ethanol concentration on the yield of total flavonoids. **(B)** The impact of the interactive effects between liquid–solid ratio and extraction time on the yield of total flavonoids. **(C)** The impact of the interactive effects between ethanol concentration and extraction time on the yield of total flavonoids.

Based on the outcomes of the preliminary single-factor tests, the highest flavonoid yields were observed at 4.02, 4.15, and 5.69% for samples 1, 2, and 3, respectively, as detailed in Supplementary Table S2. The data from Supplementary Table S2 indicate that the ultrasonic-assisted extraction efficiency of total flavonoids from *Z. bungeanum* residue was notably influenced by the sieve treatment with varying pore sizes, with the highest flavonoid content being achieved in sample 1. Consequently, sample 1 was selected for the further optimization of the extraction process using the Response Surface Methodology. In this optimization process, a three-factor, three-level experiment was meticulously planned employing the Box–Behnken design. This design facilitated the execution of 17 experiments, each with a unique combination of factors aimed at optimizing the collective impact of the liquid-to-solid ratio (X1), ethanol concentration (X2), and extraction time (X3). A quadratic polynomial equation was determined to accurately model the influence of the aforementioned factors on the yield of total flavonoids from *Z. bungeanum* residue. The resulting regression equation is as follows: Y = 3.95–0.0758 × X_1_ + 0.1543 × X_2_–0.0836 × X_3_–0.0732 × X_1_X_2_–0.1643 × X_1_X_3_ + 0.0384 × X_2_X_3_–0.0963 × X_1_^2^–0.3846 × X_2_^2^–0.0870 × X_3_^2^. Meanwhile, an Analysis of Variance (ANOVA) was conducted to evaluate the statistical significance of the model, as presented in Supplementary Table S3. The ANOVA results indicated that the regression model is highly significant, with a *p*-value of 0.0014 (*p* < 0.01) and an *F*-value of 12.750. This suggests that the model can account for 99.86% of the variability in the experimental data within the tested range. Additionally, the lack of fit was not significant (*p* > 0.05), indicating a strong model fit with minimal experimental error. Subsequently, three-dimensional response surfaces and two-dimensional contour plots were generated for the factors X1, X2, and X3 to visually assess the interactive effects on the yield of total flavonoids (Figure 2), and the order of influence of the aforementioned factors on the yield of total flavonoids from *Z. bungeanum* residue was determined to be: X2 > X3 > X1. Moreover, the interaction effects between any two of these factors were not statistically significant. Meanwhile, the highest yield of total flavonoids was increased to 4.08% when the extraction conditions were a liquid-to-solid ratio of 10:1, ethanol concentration of 60%, and extraction time of 30 min (Supplementary Table S4).

### 3.3 *Zanthoxylum bungeanum* residue extract presented potential as novel algicidal agents for the management of algal blooms

The Fv/Fm ratio, maximum quantum yield of photosystem II (PSII), is an essential parameter for assessing the health and functionality of the photosynthetic system, especially under different environmental stresses. Typically, a higher Fv/Fm ratio is associated with robust plant growth, and a reduction in Fv/Fm value indicates significant stress responses in plants, correlating with physiological changes such as decreased photosynthetic rates and increased oxidative damage (Koyukan et al., 2024). Given the rapid detection characteristic of prompt fluorescence emitted by Chl *a*, the Fv/Fm value was used to reflect the growth state of algal cells under the addition of *Z. bungeanum* residue extract.

**Figure 3:**
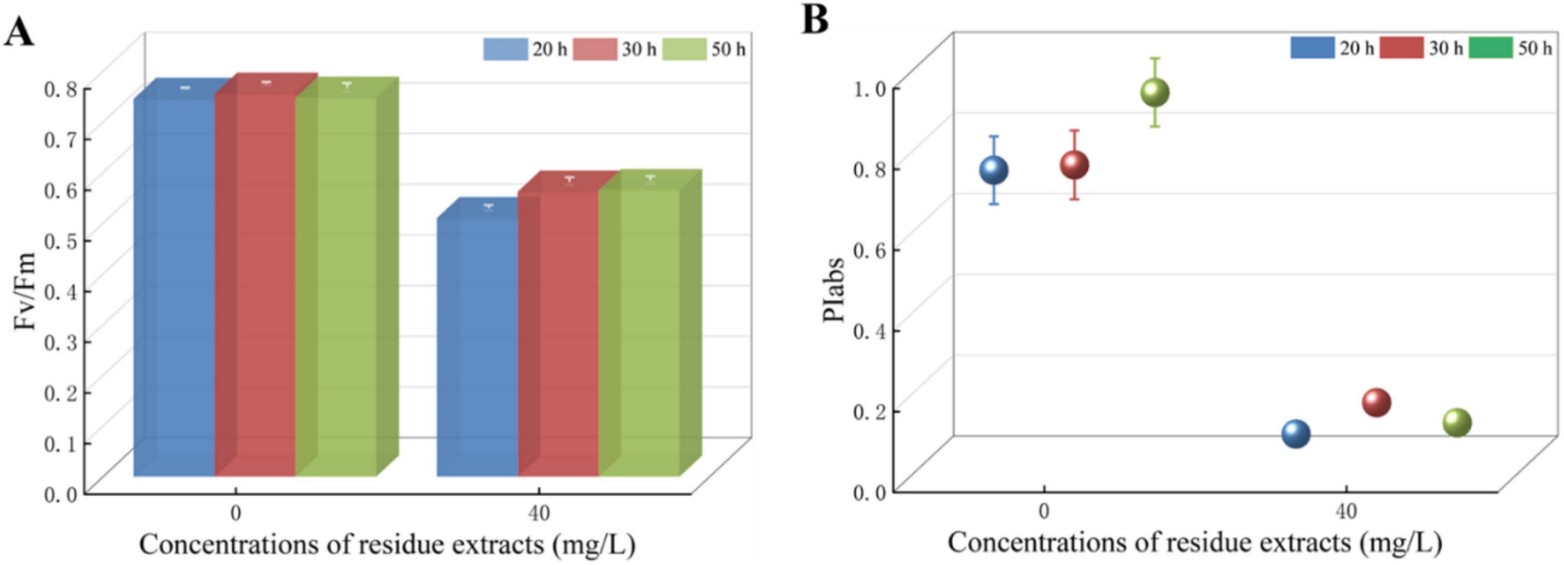
The values of Fv/Fm **(A)** and PIabs **(B)** of *T. obliquus* under *Z. bungeanum* residue extract treatment and control conditions. Significant difference between treatment group and control group was observed. Values represent the mean of three independent measurements and bars indicate SD. All processes were biologically repeated in three independent and parallel experiments.

In this study, 60% ethanol concentration was chosen as the optimum extraction solvent of total flavonoids from *Z. bungeanum* residue. *T. obliquus* with equal initial cell densities was exposed to *Z. bungeanum* residue extract in concentration of 40.0 mg/L, and the ethanol concentration in the culture medium at treatment group was less than 1%. We firstly studied the effect of 1% ethanol on *T. obliquus*, and there was no significant difference in Fv/Fm value among *T. obliquus* cells cultured at 1% ethanol and without ethanol, indicating 1% ethanol had no significant effect on the growth of *T. obliquus* (Data not shown). Subsequently, various volumes of *Z. bungeanum* residue extract solutions were introduced to these cultures, resulting in the concentrations of 0.0 and 40.0 mg/L. Interestingly, the Fv/Fm value of *T. obliquus* with 40 mg/L total flavonoids equivalent treatment was significantly lower compared to the control group over the 50-h test period (*p* < 0.05). Specifically, the Fv/Fm value of the algae cells treated for 20 h decreased by 31.68% compared to the control group, however, the inhibition rate of Fv/Fm value slightly increased with the prolongation of processing time. When the processing time was 30 h and 50 h, the inhibition rate of Fv/Fm values in treated cells decreased by 25.56 and 24.36%, respectively (Figure 3A). These results suggested that the *Z. bungeanum* residue extract notably inhibited the photosynthetic rates of *T. obliquus* during the 50-h test period. Meanwhile, *Z. bungeanum* residue extract induced a decrease in PIabs value, which was used to quantify the behavior of PSII (Figure 3B). These results showed that the PSII reaction centers have been damaged under *Z. bungeanum* residue extract stress, and PIabs was more sensitive to external threats.

Previous study has demonstrated that *Z. bungeanum* has a range of biological activities, including insecticidal, antiviral, antifungal, antioxidant, antitumor, anti-hypertension, anti-inflammatory, and antimicrobial effects (Chen et al., 2018). And a number of active compounds have been identified in *Z. bungeanum*, such as flavonoids, terpenoids, alkaloids, etc. (Zhang et al., 2017). Flavonoids, natural polyphenolic chemicals, are crucial in photosynthetic cell metabolism and exhibit diverse biological properties, notably antibacterial effects (Cushnie and Lamb, 2005; Havsteen, 1983). However, the potential of *Z. bungeanum* residue for alga control has not been fully studied, which may provide a great prospect for the development of antimicrobial agents for the management of algal blooms. As a proof of concept, it was demonstrated that photosynthetic rates of *T. obliquus* can be inhibited by *Z. bungeanum* residue extracts, which have been reported to contain abundant flavonoids (Cheng J. et al., 2024). Consequently, the yield of total flavonoids from *Z. bungeanum* residue should be optimized to further evaluate the feasibility of industrial application, and allelopathic inhibitory mechanism on the photosynthetic activity of *T. obliquus* should be further identified in the following research.

### 3.4 Effects of *Zanthoxylum bungeanum* residue extract on photosynthetic activity of *Tetrodesmus obliquus*

Photosystem II (PSII) is acknowledged as the most sensitive component within the photosynthetic apparatus. This heightened sensitivity can result in substantial decreases in photosynthetic capacity, particularly when plants are subjected to allelochemicals (Zhu et al., 2021). The prompt fluorescence emitted by chlorophyll *a* (Chl *a*) serves as a mirror of the plant’s physiological state. The fluorescence rise kinetic curves, known as OJIP transients, trace the increase in chlorophyll fluorescence from the initial O level (representing the dark-adapted state) to the peak I level (indicating maximum fluorescence). These curves are reflective of the efficiency of energy conversion processes within PSII. Analysis of the OJIP transients allows for the assessment of various parameters, which together provide a comprehensive indication of the health and functionality of the photosynthetic machinery (Guo et al., 2020).

**Figure 4:**
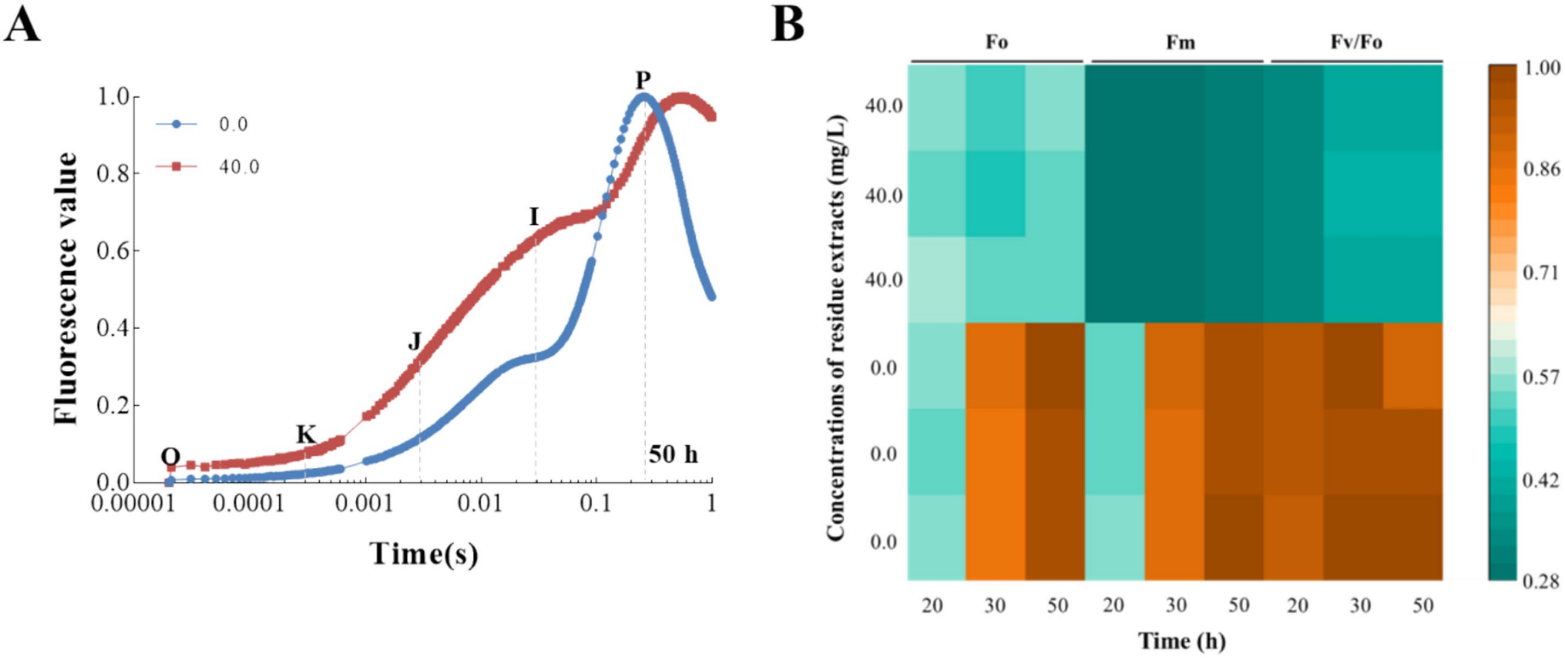
The effects of *Z. bungeanum* residue extract on the OJIP transients of *T. obliquus*. **(A)** Raw Chl *a* fluorescence rise kinetics of *Z. bungeanum* residue extract-treated *T. obliquus* cells and the control in a logarithmic time scale. The marks indicate the time points with O (at 20 μs), K (at 300 μs), J (at 2 ms), I (at 30 ms), and *p* (at the time of the maximal fluorescence intensity), which could be used in the JIP-test for the calculation of structural and functional parameters. **(B)** Effects of *Z. bungeanum* residue extract on Fo, Fm and Fv/Fo values. All the values are normalized, respectively, to that of the control at 50 h. All processes were biologically repeated in three independent and parallel experiments. Fo, minimal fluorescence intensity at 20 μs; Fm, maximum fluorescence intensity; Fv/Fo, PSII photochemical parameter.

In our research, we observed that the extract had a significant inhibitory effect on the photosynthetic rates of *T. obliquus*, attributed to a decrease in the Fv/Fm and PIabs values as depicted in Figure 3. This aligns with our expectations, as the extract also showed a substantial impact on the fluorescence transients when compared to the control group, as illustrated in Figure 4. Notably, the well-known fluorescence rise characteristic curve was observed in all groups. However, the treatment group exhibited a significant increase in the levels of the J-step and I-step during the cultivation cycle, surpassing those of the control group (Figure 4A). Meanwhile, the Fo value in the treatment group showed a 44.10% reduction compared to the control, indicating that the *Z. bungeanum* residue extract induced photo-oxidative damage in *T. obliquus* (Figure 4B). This damage is hypothesized to be linked to alterations in the structural integrity and organization of the light-harvesting complexes.

**Figure 5:**
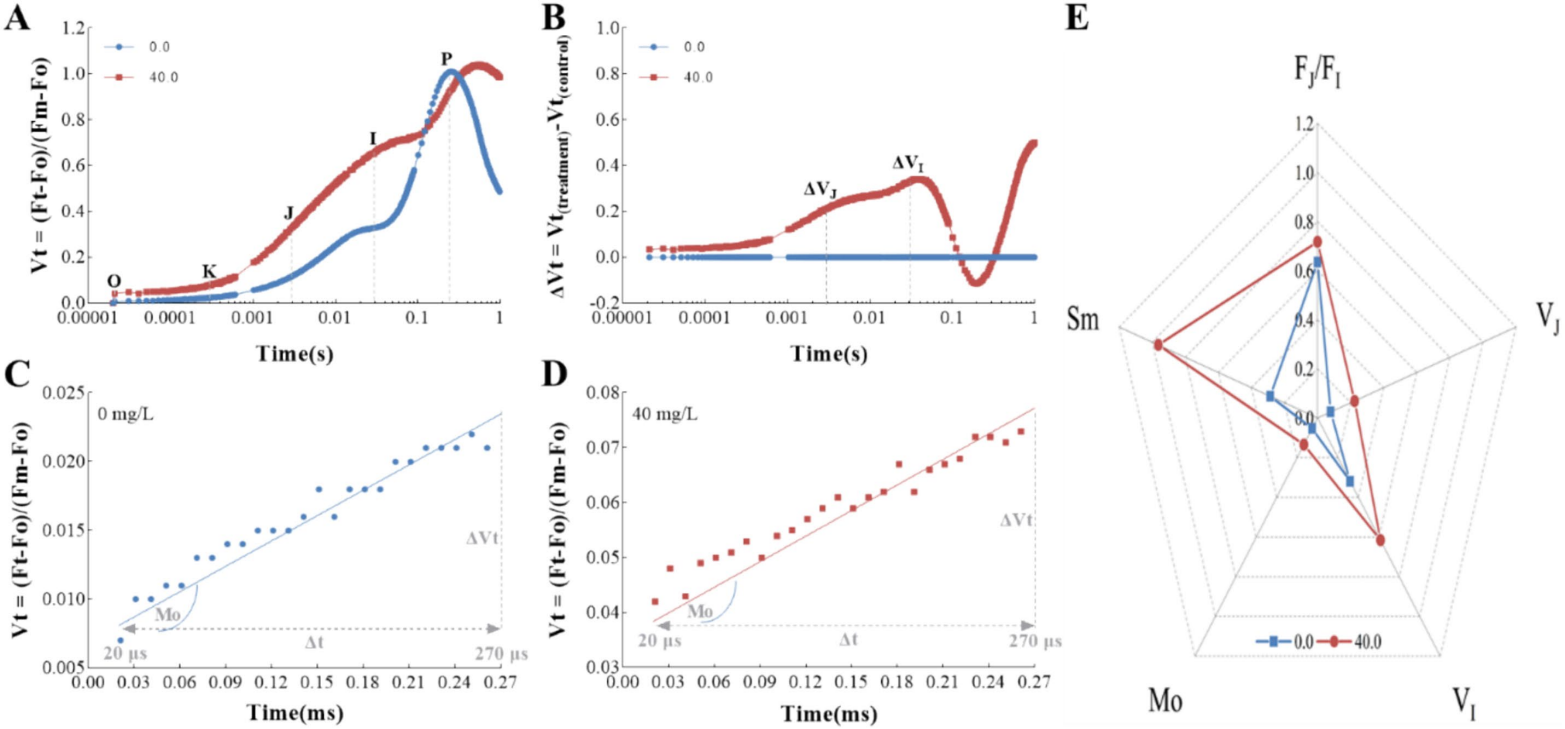
The effects of *Z. bungeanum* residue extract on the relative variable fluorescence calculated by double normalized between Fo and Fm values in the fluorescence curves. **(A)** Differences in the shapes of OJIP transient curves measured in *T. obliquus* exposed to *Z. bungeanum* residue extract. **(B)** Differences in the amplitudes of OJIP transient curves measured in *T. obliquus* exposed to *Z. bungeanum* residue extract. **(C)** The relative variable fluorescence Vt vs. Time, from 20 μs to 270 μs in a linear time scale to show the initial slope at the control group. **(D)** The relative variable fluorescence Vt vs. Time, from 20 μs to 270 μs in a linear time scale to show the initial slope at the treatment group. **(E)** Radar plot of V_J_, V_I_, Mo, Sm, and F_J_/F_I_ under *Z. bungeanum* residue extract treatment and control conditions. Mo, approximated initial slope of relative variable fluorescence Fv; Sm, normalized total complementary area above the OJIP curve; V_J_, the relative variable fluorescence at 2 ms; V_I_, the relative variable fluorescence at 30 ms; F_J_, the fluorescence intensity at 2 ms; F_I_, the fluorescence intensity at 30 ms. All processes were biologically repeated in three independent and parallel experiments.

The relative variable fluorescence (Vt) was determined by double normalization between the Fo and Fm values in the fluorescence curves, providing further insights into the characteristics of the OJIP transient (Figure 5). Notably, treatment with the *Z. bungeanum* residue extract led to a significant increase in the J-step, which is crucial for the accumulation of Q_A_^−^ (Figures 5A,B). The accumulation of Q_A_^−^, the reduced form of the primary quinone acceptor Q_A_, is likely due to an increased reduction rate of Q_A_. To delve deeper into this observation, we assessed the reduction rate of Q_A_ by analyzing the approximated initial slope of the OJIP transients. The Mo value, which represents this rate, was found to be higher in samples treated with the *Z. bungeanum* residue extract compared to untreated samples (Figures 5C,D). These results suggest that the extract enhances the net rate of reaction center closure, potentially due to the inactivation of the reaction center as a result of Q_A_^−^ accumulation. Additionally, the accumulation of Q_A_^−^ indicates that the *Z. bungeanum* residue extract could inhibit electron flow beyond Q_A_, as reported by previous research (Strasser et al., 2004). To pinpoint the initial site of action of the extract, we analyzed the relative variable fluorescence at the J-step (V_J_) and the I-step (V_I_). As shown in Figure 5E, the V_J_ and V_I_ values in samples treated with the extract were 1.84-fold and 0.93-fold higher, respectively, than in untreated samples. This led to a significant increase in the F_J_/F_I_ ratio, strongly suggesting that the J-step is the initial site of action where the extract exerts its effect.

**Figure 6:**
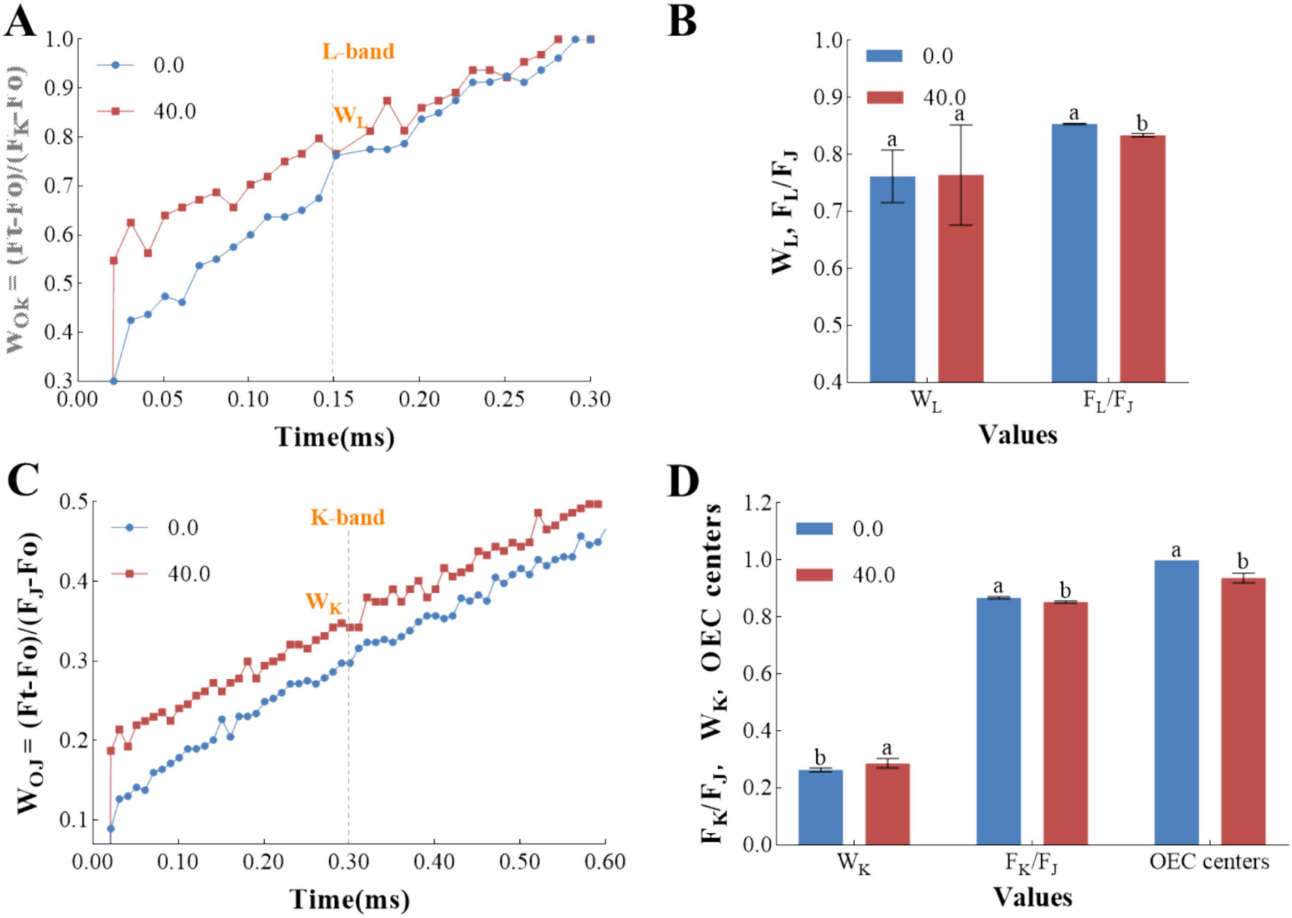
The effects of *Z. bungeanum* residue extract on the L-band and K-band. **(A)** The normalization of the OJIP transient curves calculated by double normalized between Fo and F_K_ values in the fluorescence curves in a linear time scale from 0 to 300 μs. **(B)** The values of W_L_ and F_L_/F_J_ under *Z. bungeanum* residue extract treatment and control conditions. **(C)** The normalization of the OJIP transient curves calculated by double normalized between Fo and F_J_ values in the fluorescence curves in a linear time scale from 0 to 600 μs. **(D)** The values of W_K_, F_K_/F_J_ and OEC centers under *Z. bungeanum* residue extract treatment and control conditions. Ft, the fluorescence intensity at different time points; Fo, minimal fluorescence intensity at 20 μs; F_K_, the fluorescence intensity at 300 μs; F_J_, the fluorescence intensity at 2 ms; F_L_, the fluorescence intensity at 150 μs; W_L_ = (F_L_-Fo)/(F_K_-Fo); W_K_ = (F_K_-Fo)/(F_K_-Fo); V_K_, the relative variable fluorescence at 300 μs; V_J_, the relative variable fluorescence at 2 ms; OEC centers = [1–(V_K_/V_J_)]_treatment_/[1–(V_K_/V_J_)]_control_. All processes were biologically repeated in three independent and parallel experiments. Values represent the mean of three independent measurements and bars indicate SD, and different letters indicate a significant difference at the 0.05 level (*p* < 0.05, Duncan’s multiple range test).

The differential curves for the L-band and K-band are instrumental in providing a nuanced analysis of the primary photochemical states (Strasser et al., 2007). The L-band, in particular, offers insights into the energetic connectivity of PSII units (Kalaji et al., 2018; Yusuf et al., 2010). As depicted in Figure 6A, the presence of a positive L-band in samples treated with the *Z. bungeanum* residue extract indicates a reduction in the grouping of PSII units and the energetic connectivity between the antenna and the PSII reaction centers. Consequently, this leads to a deceleration in the energy transfer from the antenna complexes to the reaction centers. These findings support the conclusion that treatment with the *Z. bungeanum* residue extract results in a diminished capacity for light absorption and utilization during the initial stages of photosynthesis. This reduction may be due to the compromised integrity of the thylakoid membrane structure following prolonged exposure to stress conditions (Gajdosik et al., 2022). Furthermore, samples treated with the *Z. bungeanum* residue extract showed a slight increase in W_L_ values. However, the F_L_/F_J_ ratio in these treated samples exhibited a significant decrease compared to the control, which can be attributed to an elevation in the J-step (Figure 6B). This suggests that the treatment affects the efficiency of energy transfer at specific steps within the photosynthetic process.

Furthermore, the OJIP transients were normalized with respect to the O-step and J-step to assess the impact of the *Z. bungeanum* residue extract on the K-step, where the K-band serves as an indicator of the active oxygen-evolving complex (OEC) centers at the PSII donor side (Yusuf et al., 2010). Figure 6C illustrates that the *Z. bungeanum* residue extract induced the appearance of the K-band, signifying the inactivation of OEC centers at the PSII donor side and an enlargement of the functional PSII antennae size in the treated samples (Yusuf et al., 2010). Meanwhile, the W_K_ value in samples treated with the *Z. bungeanum* residue extract was 9.13% higher than that of the control (*p* < 0.05), while the OEC centers value was 6.30% lower in cells exposed to the extract compared to those in the extract-free medium (*p* < 0.05). These findings suggest that the *Z. bungeanum* residue extract can cause structural damage to the OEC (Figure 6D). Additionally, the pronounced K-band in the treatment group indicates that the extract can disrupt the intracellular electron donation balance from OEC to the oxidized chlorophyll at the PSII reaction centers due to the uncoupling of OEC (Dabrowski et al., 2016; Kalaji et al., 2018). It is noteworthy that the F_K_/F_J_ value in the treated sample exhibited a significant decrease compared to the control due to an elevation in the J-step (*p* < 0.05), and the inhibition of PSII electron flow beyond Q_A_ caused by the rise of J-step will lead to electron leakage, which will contribute to the formation of reactive oxygen species. Consequently, the heightened L-steps and K-steps might be indicative of oxidative damage.

**Figure 7:**
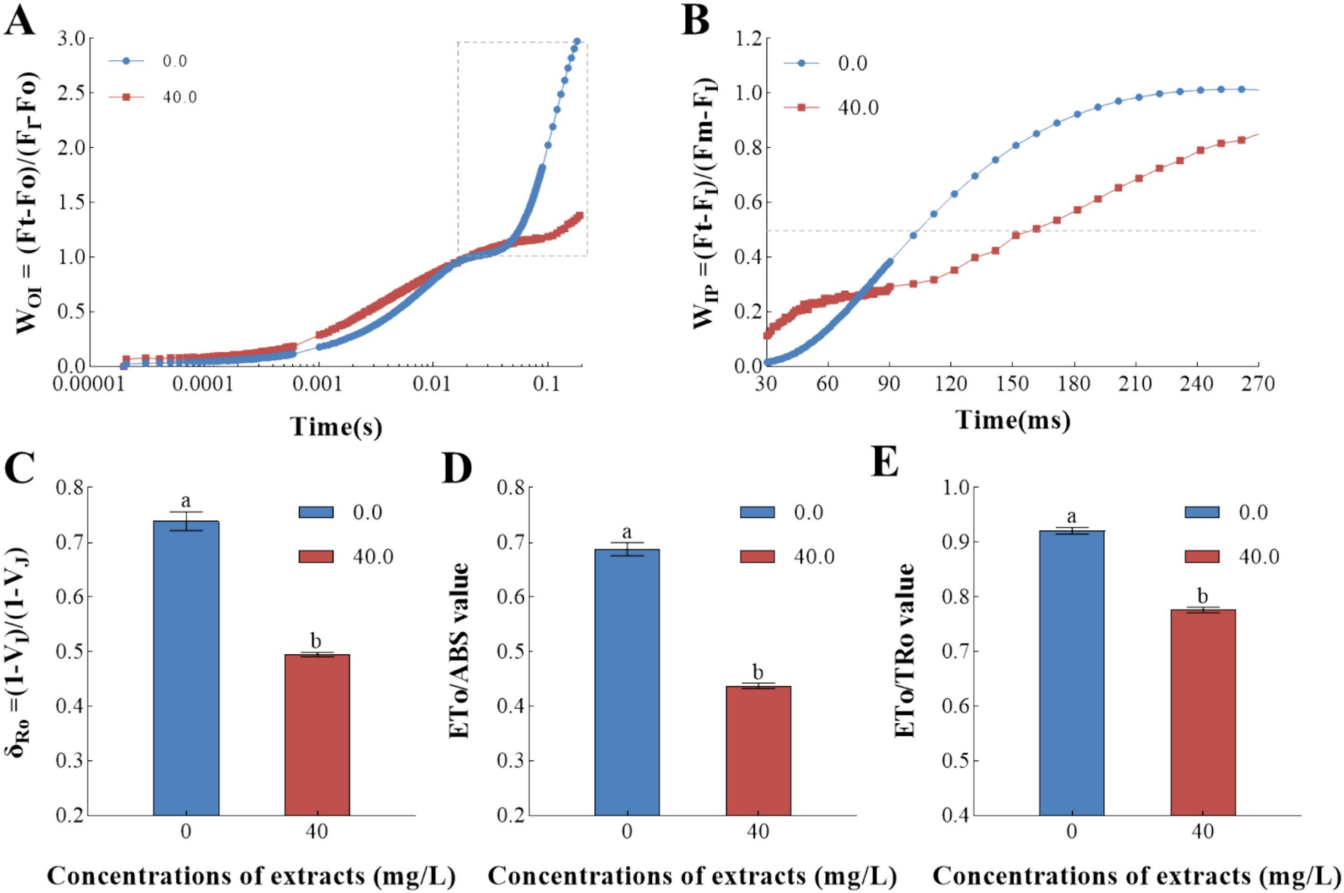
Electron transport efficiency of *Z. bungeanum* residue extract-treated cells and control. **(A)** The OJIP transient curves normalized by Fo and F_I_ values in a logarithmic time scale. **(B)** The OJIP transient curves normalized by F_I_ and Fm values in a time scale from 30 to 270 μs. **(C)** The probability that an electron is transported from the reduced intersystem electron acceptors to final electron acceptors of PSI (δ_Ro_) under *Z. bungeanum* residue extract treatment and control conditions. **(D)** Quantum yield for electron transport (ETo/ABS) under *Z. bungeanum* residue extract treatment and control conditions. **(E)** Probability that a trapped exciton moves an electron further than Q_A_ (ETo/TRo) under *Z. bungeanum* residue extract treatment and control conditions. Ft, the fluorescence intensity at different time points; Fo, the minimal fluorescence intensity at 20 μs; F_I_, the fluorescence intensity at 30 ms; Fm, the maximum fluorescence intensity; V_I_, the relative variable fluorescence at 30 ms; V_J_, the relative variable fluorescence at 2 ms; ETo/TRo, the probability that a trapped exciton moves an electron into the electron transport chain beyond Q_A_; ETo/ABS, quantum yield for electron transport. All processes were biologically repeated in three independent and parallel experiments. Values represent the mean of three independent measurements and bars indicate SD, and different letters indicate a significant difference at the 0.05 level (*p* < 0.05, Duncan’s multiple range test).

The OJIP transients were also normalized by O-step and I-step to investigate the effects of *Z. bungeanum* residue extract on the pool size of the end electron acceptors in photosystem I (PSI) acceptor side. As shown in Figures 7A, a decrease in the amplitude of W_OI_ curves was obtained in treated sample, and half rise-time values of *Z. bungeanum* residue extract-treated sample at 160 ms were greater than the control at 110 ms, indicating the reduction rates of the end electron acceptors in PSI inhibited in treated sample (Figure 7B). Meanwhile, *Z. bungeanum* residue extract treatment decreased significantly the value of δ_Ro_. Specifically, the δ_Ro_ value was reduced to 33.02% of that in the control group, demonstrating the size and functionality of PSI acceptor pool decreased (Figure 7C). At this time, the electron transfer impediment from the reduced electron acceptors to final electron acceptors of PSI have been also obtained, which can be verified by the decrease of ETo/ABS and ETo/TRo values (Figures 7D,E).

**Figure 8:**
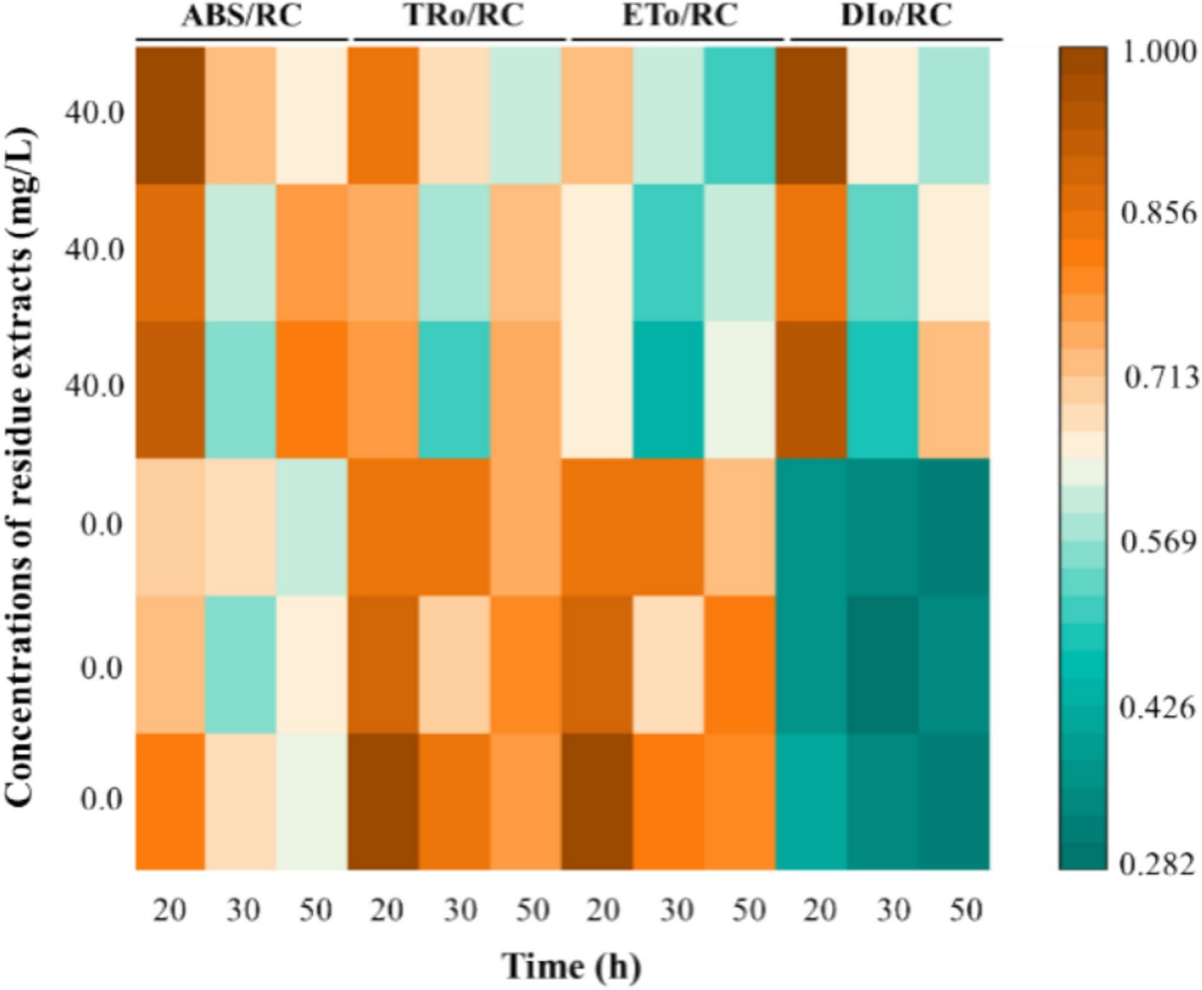
The primary photosynthetic processes estimated by the energy flux ratios per active reaction centers under *Z. bungeanum* residue extract treatment and control conditions at various time intervals (20, 30, and 50 h). ABS/RC, absorption flux per active reaction center; TRo/RC, trapping flux per active reaction center; ETo/RC, electron transport flux per active reaction center; DIo/RC, dissipation flux per active reaction center. All processes were biologically repeated in three independent and parallel experiments.

Plants under external stresses adapt to their environments by adjusting their energy distribution, primarily preventing photoinhibition (Gautam et al., 2014; Zushi et al., 2012). Hence, the effects of *Z. bungeanum* residue extract on primary photosynthetic processes was estimated by the energy flux ratios per active reaction centers (Figure 8). In this study, a significant increasing trend of ABS/RC and DIo/RC was observed, and the ABS/RC value in treated sample was 18.58% higher than that of the control group. However, *Z. bungeanum* residue extract induced a decrease in ETo/RC and TRo/RC values. There was a possibility that the formation of Q_A_-non-reducing reaction centers in treated sample can efficiently absorb light energy, but cannot be used for reduction Q_A_, resulting in excessive energy being released in the form of thermal dissipation, which can be verified by the increase of DIo/RC value (Strasser et al., 2004; Yusuf et al., 2010). Previous research has shown that allelochemicals can significantly alter the energy kinetics, which was consistent with our findings (Zhu et al., 2010). The above results suggested that formation of a protective mechanism have been induced in treated cells to resist photooxidative damage, which may represent a self-protection mechanism of algal cells against environmental stresses (Kalaji et al., 2014). Meanwhile, algae are capable of generating reactive oxygen species (ROS) under adverse conditions, and these ROS generated via the electron transport chain may result in oxidative damage (Lopes et al., 2022; Zhao et al., 2024).

The above findings contribute to a deeper understanding of the allelopathic mechanisms of *Z. bungeanum* residue extract and offer valuable insights for the management of algal blooms. However, this study is carried out under experimental conditions, and the application and analysis of *Z. bungeanum* residue extract on mixed algae in actual water bodies should be increased in the following research. Moreover, the preparation of sustained-release microspheres from *Z. bungeanum* residue extract coupled with carrier materials can continuously induce allelochemical effects to achieve long-term inhibition on bloom-forming algae, and long-term ecological impacts should also be evaluated during field validation. Anyway, *Z. bungeanum* residue extract could be served potential as novel algicidal agent to solve the harmful algae blooms in the future.

## 4 Conclusion

In this study, the ability of *Z. bungeanum* residue extract to control algal blooms has been firstly verified. Then a three-factor, three-level Box–Behnken design was used to determine the optimal yield of the primary antimicrobial component, flavonoids to assess the feasibility of industrial application. The effects of *Z. bungeanum* residue extract on the photosynthetic activity of *T. obliquus* and the underlying physiological mechanisms were investigated. The optimal extraction conditions for achieving a total flavonoids yield of 4.08% were identified as liquid-to-solid ratio of 10:1, ethanol concentration of 60%, extraction temperature of 80°C, and extraction time of 30 min. Meanwhile, OJIP transients indicated that the J-step was the initial site of action, and extract induced damage at multiple levels of physiological and biochemical processes in microalgal cells, including decrease in electron transport capability, inhibition in energetic connectivity between antenna and PSII reaction centers, incomplete in the structure of OEC, and disruption of energy distribution in PSII reaction center. The optimized extraction conditions obtained in this study are the cost-effective process for large-scale applications, and the preliminary analysis of the extract’s effects on photosynthetic activity could be used as a foundation for potential industrial applications in controlling algal proliferation.

## Data Availability

The original contributions presented in the study are included in the article/Supplementary material, further inquiries can be directed to the corresponding authors.
