# Supplementary material for: Optimization of ultrasonic-assisted extraction of total flavonoids from Zanthoxylum bungeanum residue by response surface methodology and evaluation of its algicidal properties
Source: Front Microbiol. 2025 Mar 13;16:1540631. doi: 10.3389/fmicb.2025.1540631 (PMC11966038; doi:10.3389/fmicb.2025.1540631)
Supplement: Supplementary file 1 [file Table_1.docx]

**Supplementary Information**

**Optimization of Ultrasonic-Assisted Extraction of Total Flavonoids from *Zanthoxylum bungeanum* residue by Response Surface Methodology and Evaluation of Its Algicidal Properties**

Jie Cheng ^1,^*, Long Tan ^1^, Yucai Wang ^1^, Mengwei Gao ^1^, Feifei Liu ^1^, Qi Wang ^1^, Chengshuai Xu ^1^, Chaobo Zhang ^1^, Wei Xu ^1^, Yuyong Hou ^2,^*, Tong Jiang ^3^, Lei Zhao ^2,^*

^1^ State Key Laboratory of Macromolecular Drugs and Large-scale Preparation, School of Pharmaceutical Sciences and Food Engineering, Liaocheng University, Liaocheng 252000, China;

^2^ Key Laboratory of Engineering Biology for Low-carbon Manufacturing, Tianjin Institute of Industrial Biotechnology, Chinese Academy of Sciences, Tianjin 300308, China;

^3^ College of Agriculture and Biology, Liaocheng University, Liaocheng 252000, China.

* Corresponding authors:

zhaol@tib.cas.cn (L. Zhao); hou_yy@tib.cas.cn (Y. Hou); chengjie@lcu.edu.cn (J. Cheng)

**Table S1** Factors and levels of response surface experiments

| Factors | Levels | | |
| --- | --- | --- | --- |
|  | -1 | 0 | 1 |
| Liquid-to-solid ratio (X1, mL/g) | 5:1 | 10:1 | 15:1 |
| Ethanol concentration (X2, %) | 40 | 60 | 80 |
| Extraction time (X3, min) | 25 | 35 | 45 |

**Table S2** The optimal flavonoid content in samples 1, samples 2, and samples 3 by the single factor tests

| Samples | Sample1 | Sample2 | Sample3 |
| --- | --- | --- | --- |
| Mass before sifting / g | 200.00 | 200.00 | 200.00 |
| Mass after sifting / g | 159.50 | 113.33 | 36.86 |
| Flavonoids yield / % | 4.02 | 4.15 | 5.69 |
| Flavonoids content / g | 6.415 | 4.703 | 2.098 |

**Table S3** ANOVA for the regression quadratic model equation of Box–Behnken Design

| Source | Sum of Squares | df | Mean Square | F-value | P-value |
| --- | --- | --- | --- | --- | --- |
| **Model** | 1.160 | 9 | 0.129 | 12.750 | 0.001** |
| X_1_ | 0.046 | 1 | 0.046 | 4.540 | 0.070 |
| X_2_ | 0.191 | 1 | 0.191 | 18.810 | 0.003* |
| X_3_ | 0.056 | 1 | 0.056 | 5.520 | 0.050* |
| X_1_X_2_ | 0.021 | 1 | 0.021 | 2.120 | 0.189 |
| X_1_X_3_ | 0.108 | 1 | 0.108 | 10.67 | 0.010* |
| X_2_X_3_ | 0.006 | 1 | 0.006 | 0.583 | 0.470 |
| X_1_^2^ | 0.039 | 1 | 0.039 | 3.850 | 0.090 |
| X_2_^2^ | 0.623 | 1 | 0.623 | 61.480 | ＜0.001** |
| X_3_^2^ | 0.032 | 1 | 0.032 | 3.150 | 0.119 |
| **Residual** | 0.071 | 7 | 0.010 |  |  |
| Lack of Fit | 0.033 | 3 | 0.011 | 1.140 | 0.434 |
| Pure Error | 0.038 | 4 | 0.010 |  |  |
| **Cor Total** | 1.230 | 16 |  |  |  |

Note: df, the degree of freedom; F-value refers to the F statistic obtained by performing an F test; P-value refers to probability and is relevant to significance.

**Table S4** Box–Behnken Design for the independent variables and corresponding response values

| **Run order** | **X_1_** | **X_2_** | **X_3_** | **Response Value** |
| --- | --- | --- | --- | --- |
| 1 | -1 | -1 | 0 | 3.32 |
| 2 | 1 | -1 | 0 | 3.30 |
| 3 | 0 | 0 | 0 | 3.86 |
| 4 | 1 | 1 | 0 | 3.48 |
| 5 | -1 | 0 | -1 | 3.69 |
| 6 | 1 | 0 | -1 | 3.88 |
| 7 | -1 | 0 | 1 | 3.98 |
| 8 | 0 | 0 | 0 | 4.08 |
| 9 | 0 | -1 | -1 | 3.51 |
| 10 | 0 | 1 | -1 | 3.73 |
| 11 | 0 | -1 | 1 | 3.14 |
| 12 | 0 | 1 | 1 | 3.51 |
| 13 | 0 | 0 | 0 | 3.93 |
| 14 | 1 | 0 | 1 | 3.51 |
| 15 | 0 | 0 | 0 | 4.01 |
| 16 | -1 | 1 | 0 | 3.78 |
| 17 | 0 | 0 | 0 | 3.85 |
